# The brain anatomy of attention-deficit/hyperactivity disorder in young adults – a magnetic resonance imaging study

**DOI:** 10.1371/journal.pone.0175433

**Published:** 2017-04-13

**Authors:** Jean-G. Gehricke, Frithjof Kruggel, Tanyaporn Thampipop, Sharina Dyan Alejo, Erik Tatos, James Fallon, L. Tugan Muftuler

**Affiliations:** 1 Department of Pediatrics, University of California, Irvine, Irvine, California, United States of America; 2 The Center for Autism & Neurodevelopmental Disorders, Santa Ana, California, United States of America; 3 Department of Biomedical Engineering, University of California, Irvine, Irvine, California, United States of America; 4 Department of Anatomy & Neurobiology, University of California, Irvine, Irvine, California, United States of America; 5 Department of Neurosurgery, Medical College of Wisconsin, Milwaukee, Wisconsin, United States of America; University of California, San Francisco, UNITED STATES

## Abstract

**Background:**

This is one of the first studies to examine the structural brain anatomy and connectivity associated with an ADHD diagnosis and child as well as adult ADHD symptoms in young adults. It was hypothesized that an adult ADHD diagnosis and in particular childhood symptoms, are associated with widespread changes in the brain macro- and microstructure, which can be used to develop a morphometric biomarker for ADHD.

**Methods:**

Voxel-wise linear regression models were used to examine structural and diffusion-weighted MRI data in 72 participants (31 young adults with ADHD and 41 controls without ADHD) in relation to diagnosis and the number of self-reported child and adult symptoms.

**Results:**

Findings revealed significant associations between ADHD diagnosis and widespread changes to the maturation of white matter fiber bundles and gray matter density in the brain, such as structural shape changes (incomplete maturation) of the middle and superior temporal gyrus, and fronto-basal portions of both frontal lobes. ADHD symptoms in childhood showed the strongest association with brain macro- and microstructural abnormalities. At the brain circuitry level, the superior longitudinal fasciculus (SLF) and cortico-limbic areas are dysfunctional in individuals with ADHD. The morphometric findings predicted an ADHD diagnosis correctly up to 83% of all cases.

**Conclusion:**

An adult ADHD diagnosis and in particular childhood symptoms are associated with widespread micro- and macrostructural changes. The SLF and cortico-limbic findings suggest complex audio-visual, motivational, and emotional dysfunctions associated with ADHD in young adults. The sensitivity of the morphometric findings in predicting an ADHD diagnosis was sufficient, which indicates that MRI-based assessments are a promising strategy for the development of a biomarker.

## Introduction

Attention-Deficit/Hyperactivity Disorder (ADHD) is characterized by inattention, hyperactivity, and impulsivity. It is one of the most frequently diagnosed neurodevelopmental disorders with a prevalence rate of approximately 5% in children and 2.5% in adults [1]. Estimates suggest that 65% of children with ADHD continue to have problems in adulthood [2]. While ADHD is well-characterized at the behavioral level in children, little is known about the association between brain structure and ADHD diagnosis as well as symptoms across the lifespan.

The most frequently used approaches to measure brain structures are based on Magnetic Resonance Imaging (MRI). T1-weighted anatomical imaging is used to assess the brain macrostructure, such as the volume and structure of white matter (i.e., a high proportion of myelinated axons) and grey matter (i.e., a high proportion of neuronal cell bodies), while diffusion-tensor imaging (DTI) is a more recent approach to specifically assess the brain microstructure. DTI provides information about the mobility of water molecules in white matter via the scalar parameters: fractional anisotropy (FA)–directionality of water diffusion perpendicular to white matter fibers [3]; radial diffusivity (RD)–diffusion rate perpendicular to white matter tracts; axial diffusivity (AD)–diffusion rate parallel to axons; and mean diffusivity (MD), reflecting overall water mobility [4, 5]. Decreases in FA can be associated with increases in MD, which suggests increased extracellular space or decreased axonal density [4]. Alternatively, decreased FA may result from increased RD, indicating decreased myelination [4, 6].

A number of studies documented the macro- and microstructural pathophysiology associated with an ADHD diagnosis. Initial brain macrostructural studies revealed that children and adolescents with ADHD show smaller brain volumes in all regions [7]. However, subsequent meta-analyses [8–11] mainly report differences in basal ganglia together with changes in total brain [8] or gray matter volume [11]. Intriguingly, most volume differences appear to normalize with transition to adulthood [12].

Besides these macrostructural indicators, it was suggested that ADHD primarily involves the fronto-striatal [13] as well as fronto-parietal networks [14]. More specifically, dysregulated functional communications in these networks are assumed to cause the behavioral dysfunction associated with ADHD [15]. A meta-analysis corroborated this notion by revealing that children with ADHD show compromised white matter (WM) integrity in the inferior and superior longitudinal fasciculus, anterior corona radiate, cortico-spinal tract, cingulum, corpus callosum, and the internal capsule and cerebellum [16]. Such compromised WM integrity may contribute to the dysfunctional communication in frontal-striatal and parietal networks. In addition, it was suggested that the pathophysiology of ADHD includes a dysregulated modulation of cortical plasticity during brain development, resulting in an abnormal cortico-cortical connectivity that may persist into adulthood [17]. Some studies support that ADHD is associated with lifelong abnormal connectivity by showing that adults with ADHD have reduced connectivity in the orbito-medial prefrontal cortex, right anterior cingulate, right anterior corona radiata and the superior as well as inferior longitudinal fasciculus among other regions [18–21], and increased MD in the orbito-medial prefrontal cortex, right anterior cingulate, and left fronto-occipital fasciculus [19, 22]. These areas are central for connecting the prefrontal cortex to the basal ganglia, consisting of caudate and putamen [23], which are smaller in children but not in adults with ADHD [7, 10].

It was suggested that changes in the striatum and its connections are associated with hyperactivity-impulsive symptoms in children with ADHD [16]. However, the size of basal ganglia regions may initially correlate with hyperactive-impulsive symptoms, which decline over time and may disappear during adulthood [24]. This notion was corroborated by showing that the lack of differences in the striatal volumes in adults with ADHD may be caused by reductions in hyperactive-impulsive symptoms in adulthood [25].

In children and adolescents, severity of ADHD symptoms was linked to decreased frontal and temporal grey matter, caudate and cerebellar volumes, [7] and decreased FA [15, 21, 26]. These findings suggest that the prefrontal cortex and its connections may be associated with ADHD symptoms such as distractibility, forgetfulness, impulsivity, poor planning and hyperactivity in both children and adults with ADHD [27]. In children and adolescents with ADHD, inattention was linked to reduced brain connectivity not only in the frontal but also in the anterior cingulate, temporal, and parietal regions [28]. However, the extent of dysfunctional connectivity has yet to be determined in young adults with ADHD. Thus far, the literature suggests that a diagnosis of ADHD as well as ADHD symptoms may be associated with widespread changes in the brain macro- and microstructure. In addition, as many adults with ADHD show symptom improvements [29], the correlations between childhood symptoms and brain macro- and microstructure may weaken in adulthood due to brain maturation or improved coping skills. However, there are no studies that directly examine brain anatomical correlates of ADHD diagnosis as well as child and adult ADHD symptoms in young adults with ADHD.

The purpose of the study was to close this gap in knowledge by analyzing structural and diffusion-weighted MRI data in relation to ADHD diagnosis and the number of self-reported childhood and adult symptoms in young adults. The aim of the study was threefold: 1. to examine macro- and microstructural correlates of an ADHD diagnosis in young adults with ADHD, 2. to study associations between brain structure and child as well as adult symptoms of ADHD, and 3. to assess if the morphometric correlates of ADHD can be used as a potential biomarker to predict an ADHD diagnosis. Based on previous research showing widespread changes in the brain macro- and microstructure, it was hypothesized that an adult ADHD diagnosis is associated with frontal, basal ganglia, anterior cingulate, temporal, and parietal regions in young adults with ADHD. In addition, it was hypothesized that correlations between brain structure and ADHD symptoms in childhood are more widespread and prominent (i.e., number, size, and z-scores of significant brain regions) than ADHD symptoms in adulthood. Moreover, the morphometric findings may predict the ADHD diagnosis in more than 80% of cases, which would demonstrate that morphometric data have the potential to be used as a biomarker for the diagnosis of ADHD.

## Methods

### Participants

Seventy-two participants participated in the study of which 31 met the diagnostic criteria for ADHD according to DSM-IV [30], and 41 controls without an ADHD disorder (see Table 1). Similar to previous studies [31–34], each participant was assessed according to DSM-IV-TR criteria [30] with the Structured Clinical Interview for DSM-IV (SCID), [35] and the QUEST method [36]. Severity of ADHD symptoms was evaluated using the Assessment of Hyperactivity and Attention (AHA), which is a quantitative measure of ADHD symptomatology [37]. The AHA [37], an 18-item questionnaire, is based on DSM-IV criteria and was used to measure severity of both childhood and adult symptoms of ADHD. Although the number of controls who met DSM-VI criteria for ADHD was zero, 21 control participants had at least one ADHD symptom. However, these ADHD symptoms were to low in frequency and did not cause sufficient clinical impairment to warrant a clinical diagnosis of ADHD. Compared to controls, participants with ADHD had lower levels of employment (χ^2^ = 4.73, *p* = 0.03), more ADHD symptoms (*t* ≥ 5.39, *p* ≤ 0.0001) as well as subtype diagnoses (*χ*^2^ ≥ 18.00, *p* ≤ 0.001), and currently used more stimulant medications (*χ*^2^ ≥ 3.91, *p* ≤ 0.05).

**Table 1:** Participant Sample Characteristics.

Characteristics	ADHD (N = 32)	Control (N = 40)
Ages, Years (M ± SD)	25.31 ± 5.44	23.93 ± 3.60
Male/Female (N)	26/6	33/7
Of European Decent (%)	68.75	62.50
Education, years (M ± SD)	13.28 ± 2.70	15.20 ± 2.36
Employed (%)	56.30	80.00*
ADHD Subtype		
Predominantly Inattentive Subtype (%)	37.50	0.00***
Predominantly Hyperactive/Impulsive Subtype (%)	6.25	0.00
Predominantly Combined Subtype (%)	56.30	0.00***
Number of Childhood ADHD Symptoms (M ± SD)	14.13 ± 3.6	5.83 ± 5.81***
Number of Inattentive Symptoms (M ± SD)	7.56 ± 1.88	3.45 ± 3.20***
Number of Hyperactive/Impulsivity Symptoms (M ± SD)	6.56 ± 2.49	2.38 ± 3.04***
Number of Adult ADHD Symptoms (M ± SD)	9.56 ± 3.885	3.30 ± 3.88***
Number of Inattentive Symptoms (M ± SD)	5.13 ± 2.50	1.98 ± 2.43***
Number of Hyperactive/Impulsivity Symptoms (M ± SD)	4.44 ± 2.26	1.33 ± 1.70***
Number of Participants with Comorbidities (%)	31.25	25.00
Anxiety Disorder (%)	21.90	10.00
Eating Disorder (%)	3.13	2.50
Learning Disorder (%)	0.00	2.50
Mood Disorder (%)	9.38	5.00
Other Substance Use Dependence (%)	9.38	7.50
Current use of Stimulant Medication (%)	25.00	0.00**
Adderall (%)	15.63	0.00**
Ritalin (%)	9.38	0.00*

*p ≤ 0.05

**p ≤ 0.01

***p ≤ .001

All participants were physically healthy and had no chronic illness such as heart disease, irregular heartbeat, hypertension, diabetes, skin allergies, or skin diseases. The study was approved by the Institutional Review Board of the University of California, Irvine and written consent was obtained from each participant between July 2009 and April 2014.

### Procedure

Participants were asked to close their eyes and relax while in the MRI scanner. The duration of the scan was 20 minutes (i.e., 10 minutes to obtain T1 weighted images and another 10 minutes to obtain diffusion weighted image set).

### Imaging protocol

MRI data were acquired on a Philips Achieva 3T scanner, equipped with an 8-channel phased array coil. T1-weighted images were acquired using a Turbo Field-Echo (TFE) sequence with TR 11 ms, TE 3.7 ms, flip angle 18 degrees, 150 sagittal slices with a matrix of 240 x 240 voxels, corresponding to an isotropic resolution of 1.0 mm. Diffusion-weighted images were acquired using a diffusion-weighted spin-echo EPI sequence with TR 7.0 s, TE 80.0 ms, flip angle 90 degrees, 60 axial slices with a matrix of 116 x 112 voxels, corresponding to a resolution of 1.8 mm x 1.8 mm (in plane), 2 mm slice thickness. Thirty-two image volumes were acquired using different diffusion weighting gradient directions at b = 1000 s/mm^**2**^, one volume with isotropic gradients at b = 1000 s/mm^2^, and a reference volume without diffusion-weighting (b = 0 s/mm^2^).

### Processing of T1-weighted images

Data analysis followed the general outline of voxel-based morphometry (VBM). The BRIAN software package was used to analyze the imaging data (http://sip.eng.uci.edu). All T1-weighted MR images were inspected for acquisition-related artifacts and signs of neurological diseases. Heads were aligned with the stereotaxic coordinate system [38] and registered with the ICBM 2009c template [39], using a recent approach for nonlinear registration [40]. All registered head images were scaled for a mean intensity of 100 and averaged. The brain was extracted from the averaged head image to yield the brain template 1. Subsequently, a mask of the intracranial volume was generated from each head dataset and used to extract the intracranial space [41]. Data were corrected for intensity inhomogeneities using a newly developed technique that estimates the gain field by comparing the global and regional intensity distribution. Next, the intensity-corrected image was segmented into three probabilistic maps (roughly corresponding to: cerebrospinal fluid (CSF) grey and white matter). The volume integral of these compartments was used as an estimate of the intracranial volume (ICV). All intensity-corrected brain images were registered with template 1, and averaged to yield the brain template 2. This procedure is considered standard for generating a study-specific template (S1 Template) [42]. The first template depends on properties of the initial atlas, while the second template was generated using information from this study alone. All brain data sets were registered with template 2, and the resulting deformation field applied to the GM (WM) probabilistic map, now interpreted as GM (WM) concentration (GMC, WMC). Finally, these maps were smoothed using a Gaussian filter (*σ* = 2, FWHM of 4.7 mm) and logit-transformed. From the deformation field obtained above, the determinant of the first partial derivative (the Jacobian) was computed. This value can be used to detect local shape changes (greater than 1 for locally expanding areas, less than one for locally contracting areas, and 1 for no change, translations and rotations). Data were smoothed using a Gaussian filter (FWHM of 4.7 mm), and log-transformed. Thus, maps were obtained for the GM (WM) concentration and Jacobian (JAC) for each subject in normalized space.

### Processing of diffusion-weighted images

The BRIAN software package was used to analyze the imaging data (http://sip.eng.uci.edu). To correct for subject motion, image volumes corresponding to all gradient directions were registered with the non-diffusion weighted reference image volume using affine registration and mutual information as an image similarity metric. Then, diffusion tensors were computed from the registered diffusion-weighted images using a nonlinear procedure including anisotropic noise filtering [43]. Tensors were converted into scalar measures fractional anisotropy (FA), radial, mean and axial diffusivity (RD, MD, AD) values. The reference volume of the DTI data set was linearly registered with the T1-weighted brain image obtained above, and the resulting transformation was used to map each measure into stereotaxic space. Next, the deformation field obtained above was used to warp measures into normalized space. Finally, these maps were smoothed using a Gaussian filter (FWHM of 4.7 mm). Thus, we obtained maps for FA, RD, MD, and AD for each subject in normalized space.

### Statistical analysis

Structural MRI data (T1- weighting) and the diffusion-weighted MRI data were analyzed in relation to diagnosis (ADHD and Controls) and the number of self-reported childhood and adult symptoms. For each of the six image series above, voxel-wise linear regression models were computed, for diagnosis (Control = 0, ADHD = 1), childhood and adulthood symptoms, and gender, as well as age at time of examination as covariates. For the independent variable grey matter (white matter) concentration, computation was restricted to a mask with a corresponding probability *p* > 0.25, for all other methods to a tissue mask with a white matter probability *p* > 0.5. As result, the significance of the regression coefficient for the regressor of interest was obtained as a voxel-wise z-score. Spatial clusters above an absolute z-score threshold of 2.5 (corresponding *p*-value of 0.01) were determined and assessed for significance (*p* < 0.05) based on the theory of excursion sets in Gaussian random fields [44]. This approach corrects for the multiple comparisons by controlling the family-wise error rate, and is based on the theory of Gaussian random fields. The cluster-extent based thresholding is currently the most popular method for multiple comparisons correction of statistical maps in neuroimaging studies, and is the commonly accepted standard in the context of voxel-based morphometry. Only clusters passing the significance test were reported. A voxel-level (primary) *p*-value threshold of *p* = 0.01 and a cluster-level (secondary) *p*-value threshold of *p* = 0.05 was used, which are standard settings for this approach. Average z-scores were computed over all voxels in a given cluster that passed the significance test. The center-of-mass, extent (in mm^**3**^), peak and average z-score were computed for each cluster, and addressed an anatomical label based on Talairach coordinates using the labeled map developed by Lancaster et al. [45], and are compiled in tables.

In order to assess whether any combination of the subject-wise measures can be used to predict an ADHD diagnosis, we extracted voxel-wise measures from regions that significantly differed by the diagnostic label. For each subject and each analysis, continuous voxel-wise measures (GMC, WMC, JAC, FA, RD, MD) in significantly different regions were collected and averaged, resulting in one score per subject and analysis. Logistic regression was used to find a single score that best predicted an ADHD diagnosis. To assess the combined predictive power of all scores, we performed a principal component analysis (PCA) to reduce the dimensionality of the data, and used a linear discriminant analysis (LDA) to develop a simple classifier in the reduced space.

## Results

### ADHD diagnosis and brain structure

An ADHD diagnosis was associated with different grey (white) matter concentrations, and shape differences as computed from the Jacobian maps (JAC, see Table 2, S1, S2 and S3 Maps). The findings revealed: 1. regions of decreased grey matter in the right middle and inferior frontal gyrus, right superior and middle temporal gyrus, left caudate head, and left parahippocampal gyrus; 2. regions with increased white matter in the right frontal gyrus, right inferior frontal gyrus, right middle temporal gyrus, left and right external capsule, left internal capsule, and left parahippocampal gyrus; 3. regions with shape contractions (decreased JAC suggesting incomplete maturation) in the right middle frontal gyrus, right superior temporal gyrus, left middle temporal gyrus, right postcentral gyrus, left posterior insula, and left and right parahippocampal gyrus.

**Table 2:** Linear regression of model data DIAGNOSIS + AGE + SEX + ICV, assessing the importance of the diagnostic group on structural data.

		MNI Coordinates			Size		
Measurement/Structure		x	y	z	mm^3^	Z_max_	Z_av_
Grey Matter							
	Right Middle Frontal Gyrus	29	35	34	376	-4.047	-2.883
	Right Inferior Frontal Gyrus	52	6	21	1383	-4.512	-3.024
	Right Superior Temporal Gyrus	52	-43	7	989	-4.245	-2.910
	Right Middle Temporal Gyrus	48	-65	15	584	-3.604	-2.871
	Left Caudate Head	-10	6	1	357	-3.483	-2.823
	Left Parahippocampal Gyrus	-24	-65	9	417	-3.821	-2.868
White Matter							
	Right Frontal Sub-Gyral WM	26	32	34	536	4.084	2.863
	Right Inferior Frontal Gyrus WM	49	6	23	1970	4.615	3.023
		46	39	7	348	3.502	2.777
	Right Middle Temporal Gyrus WM	52	-47	8	548	3.622	2.837
		49	-65	15	608	3.606	2.881
	Right External Capsule WM	25	6	13	896	3.394	2.770
	Left External Capsule WM	-23	13	19	450	3.620	2.826
	Left Internal Capsule	-9	-14	7	2910	3.930	2.856
	Left Parahippocampal Gyrus WM	-24	-65	9	715	4.134	2.953
	Pons	-3	-27	-36	562	3.286	2.761
Shape							
	Right Middle Frontal Gyrus WM	33	42	-2	650	-3.033	-2.680
		22	29	-15	592	-3.132	-2.710
	Right Superior Temporal Gyrus	30	10	-24	589	-2.923	-2.631
		51	-18	3	2382	-3.203	-2.685
	Left Middle Temporal Gyrus WM	-51	-33	-10	1649	-3.139	-2.709
	Right Postcentral Gyrus	42	-24	36	1345	-3.459	-2.857
	Left Posterior Insula	-45	-14	16	2544	-3.739	-2.874
	Left Parahippocampal Gyrus	-22	-59	7	551	-3.018	-2.652
		-12	-43	5	401	-2.927	-2.674
	Right Parahippocampal Gyrus	15	-59	11	3525	-3.144	-2.683

Regions with significant differences in GM & WM concentration, and shape (JAC) are shown, with their center position, size, peak, and mean z-score. The sign of the z-scores is given relative to controls. Positive (negative) z-scores correspond to a higher (lower) value of a specific variable in the ADHD group, e.g., a negative z-score to a lower grey matter concentration.

**Fig 1:**
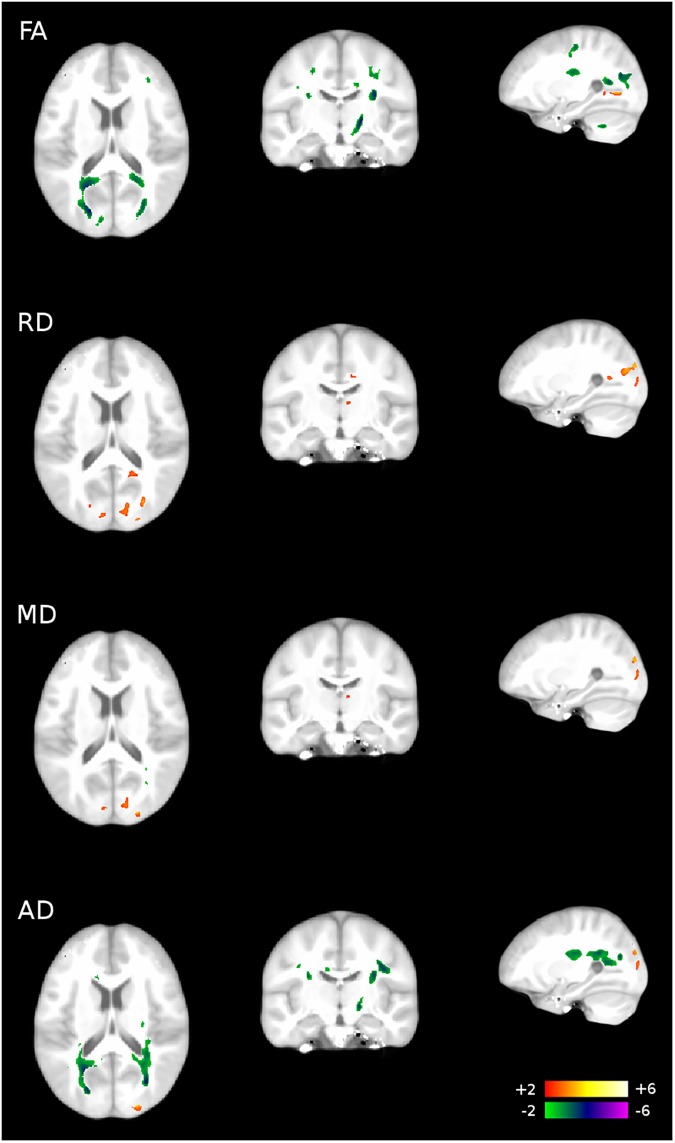
Overlay of brain areas that significantly differ in their WM properties due to ADHD diagnosis.

Table 3 (S4, S5, S6 and S7 Maps) depicts the analysis of the DTI data analysis, which showed that an ADHD diagnosis is associated with: (1) regions of increased FA in the left external capsule, as well as left and right optic radiation; (2) regions of decreased FA in the right superior temporal gyrus, left and right middle temporal gyrus, right postcentral gyrus, cingulate gyrus, corpus callosum, left and right temporal stem, and right midbrain; (3) regions of increased RD, including the left and right postcentral gyrus, left middle temporal gyrus, cingulate gyrus, right internal capsule, and right midbrain; (4) regions of decreased RD, including the left supraventricular white matter and left pons; (5) regions of primarily increased MD in the left middle temporal white matter, right internal capsule, right midbrain, and left pons; (6) regions of decreased MD in the corpus callosum and left pons; (7) regions of increased AD in the right cuneus and right middle occipital gyrus; (8) regions of decreased AD in the right precentral WM, occipital lobe, and brainstem. For an example overlay of brain areas that significantly differed in their WM properties due to ADHD, refer to Fig 1.

**Table 3:** Linear regression of model data DIAGNOSIS + AGE + SEX + ICV, assessing the importance of the diagnostic group on structural data.

		MNI Coordinates			Size		
Measurement/Structure		x	y	z	mm^3^	Z_max_	Z_av_
Fractional Anisotropy							
	Left External Capsule	-29	-3	11	1229	4.050	3.057
	Left Optic Radiation	-33	-48	-6	1120	3.552	2.865
	Right Optic Radiation	20	-59	3	1369	3.838	2.864
	Right Pons	6	-21	-30	391	3.845	3.000
	Right Superior Temporal Gyrus	35	-59	29	753	-3.654	-2.906
	Left Middle Temporal Gyrus	-42	-58	6	553	-3.996	-2.986
	Right Middle Temporal Gyrus	44	-53	6	651	-3.816	-3.074
	Right Postcentral Gyrus	42	-31	37	1093	-3.889	-2.927
	Cingulate Gyrus	9	2	34	1264	-3.021	-2.668
		14	-36	35	429	-3.021	-2.668
	Corpus Callosum	4	-39	24	384	-2.957	-2.648
		-22	-81	19	1890	-4.785	-2.981
	Sub-Gyral WM	25	-21	54	514	-3.145	-2.705
		20	-55	51	556	-3.819	-2.967
		34	-14	29	1263	-4.030	-2.926
		-29	-21	30	504	-3.265	-2.793
		34	27	29	578	-3.722	-2.954
	Left Temporal Stem	-23	-54	23	1381	-3.879	-2.992
	Right Temporal Stem	24	-66	20	1739	-3.616	-2.843
	Right Midbrain	11	-24	-4	1929	-3.974	-2.781
Radial Diffusivity							
	Left Suptraventricular WM	-21	33	37	940	-3.504	-2.769
	Left Postcentral Gyrus	-40	-32	40	763	3.731	2.864
	Right Postcentral Gyrus	43	-32	34	379	3.619	2.860
	Left Middle Temporal Gyrus	-44	-59	2	651	4.376	3.168
	Cingulate Gyrus	9	-6	39	543	3.604	2.861
	Sub-Gyral WM	-34	-66	34	480	3.264	2.751
		-20	-85	28	551	3.879	2.920
		21	-92	14	562	4.211	2.958
		18	-86	26	1512	3.959	2.893
	Right Internal Capsule	6	-8	12	534	3.028	2.690
	Right Midbrain	4	-29	-6	620	3.245	2.792
Mean Diffusivity							
	Left Middle Temporal Gyrus WM	-45	-59	1	475	4.154	3.069
	Corpus Callosum	-18	-23	29	3006	-3.260	-2.673
		33	-57	15	509	-3.286	-2.785
		-31	-58	14	663	-3.366	-2.759
		-16	-71	5	412	-3.853	-2.931
	Sub-Gyral WM	17	-88	27	1156	3.849	2.867
		-18	-86	30	464	3.364	2.777
		21	-93	15	575	4.208	2.981
	Right Internal Capsule	5	-8	12	469	3.026	2.692
	Right Midbrain	3	-29	-6	533	3.242	2.782
Axial Diffusivity							
	Right Cuneus	18	-89	29	823	3.424	2.786
	Right Middle Occipital Gyrus	21	-94	16	612	4.085	2.946
	Right Pre-central WM	34	-16	31	2417	-3.704	-2.961
	Left Occipital Lobe WM	-20	-43	26	6031	-3.707	-2.851
	Right Occipital Lobe WM	31	53	20	4041	-3.882	-2.912
	Right Brainstem	16	-22	-2	1039	-4.168	-2.893
	Left Brainstem	-16	-23	-9	361	-3.421	-2.785

Regions with significant differences in DTI measures FD, RD, MD, and AD are shown, with their center position, size, peak, and mean z-score. The sign of the z-scores is given relative to controls. Positive (negative) z-scores correspond to a higher (lower) value of a specific variable in the ADHD group.

### ADHD symptoms and brain structure

In contrast to diagnosis, no association was found between brain macrostructure and ADHD symptoms. However, ADHD symptoms were associated with microstructural findings, which were more prominent (i.e., number, size, and z-scores of significant regions) for the childhood total symptoms (see Table 4, S8, S9, S10 and S11 Maps), compared to the adult total symptoms (Table 5, S12, S13, S14 and S15 Maps). More specifically, the DTI analysis of the structural differences associated with childhood symptoms revealed: (1) a region of increased FA, RD and MD in the left sub-gyral white matter of the frontal lobe; (2) regions of increased RD and MD, including the right sub-gyral white matter of the frontal lobe and the left and right putamen and the adjacent external capsule; (3) a region of decreased RD and MD in the right superior temporal gyrus; (4) a region of decreased FA in the right putamen; (5) a region of decreased RD in the right medial frontal gyrus; (6) regions of increased AD in the right cingulate, frontal lobe, and left external capsule. The DTI analysis of the structural differences associated with adult symptoms revealed: (1) a region of decreased FA and increased RD in the right dentate nucleus; (2) a region of decreased FA, RD, MD, and AD in the left cingulum; (3) regions of increased FA, including the white matter of the right lingual gyrus, left putamen, and the white matter of the right temporo-occipital gyrus.

**Table 4:** Linear regression of model data CHILDHOOD SYMPTOMS + AGE + SEX + ICV, assessing the importance of the total number of self-reported childhood symptoms on structural data.

		MNI Coordinates			Size		
Measurement/Structure		x	y	z	mm^3^	Z_max_	Z_av_
Fractional Anisotropy							
	Left Sub-Gyral WM Frontal Lobe	-22	37	27	326	3.542	2.848
		-21	-11	51	644	3.723	2.944
		-24	1	47	415	3.523	2.887
	Right Putamen	21	0	7	458	-3.494	-2.786
Radial Diffusivity							
	Left Sub-Gyral WM Frontal Lobe	-17	29	-4	314	3.510	2.865
		-20	-10	54	328	-3.241	-2.751
	Right Sub-Gyral WM Frontal Lobe	20	-8	32	348	3.202	2.755
	Right Medial Frontal Gyrus	11	-12	64	516	-3.747	-2.888
	Right Superior Temporal Gyrus	42	4	-21	363	-3.636	-2.941
	Left Putamen/External Capsule	-27	3	9	1912	4.071	2.941
	Right Putamen/External Capsule	26	1	10	5301	4.158	3.061
Mean Diffusivity							
	Left Sub-Gyral WM Frontal Lobe	-26	6	28	1190	3.757	2.846
	Right Sub-Gyral WM Frontal Lobe	18	19	29	587	3.115	2.684
	Right Superior Temporal Gyrus WM	42	4	-20	359	-3.632	-2.994
	Left Putamen/External Capsule	-29	-24	26	2052	3.291	2.723
		-27	3	10	1687	3.693	2.833
	Right Putamen/External Capsule	26	-10	21	9069	4.269	2.875
Axial Diffusivity							
	Right Cingulate Gyrus	18	-21	46	1545	4.481	2.970
	Right Frontal Lobe WM	26	7	30	369	3.463	2.886
	Left Frontal Lobe WM	-27	5	28	551	3.726	2.854
	Left External Capsule	-31	8	11	772	3.857	2.832

Regions with significant differences in DTI measures FD, RD, MD, and AD are shown, with their center position, size, peak, and mean z-score. Positive (negative) z-scores correspond to a higher (lower) value of a specific variable in the ADHD group.

**Table 5:** Linear regression of model data ADULT SYMPTOMS + AGE + SEX + ICV, assessing the importance of the total number of self-reported adult symptoms on structural data.

		MNI Coordinates			Size		
Measurement/Structure		x	y	z	mm^3^	Z_max_	Z_av_
Fractional Anisotropy							
	Right Lingual Gyrus WM	23	-59	1	411	3.689	2.902
	Left-Cingulum WM	-14	-68	13	564	-4.225	-3.138
	Left Putamen	-28	-17	11	784	3.907	2.921
	Right Temporo-Occipital Sub-Gyral WM	38	-68	-6	416	4.058	3.113
	Right Dentate Nucleus	3	-52	-26	1889	-4.695	-3.063
Radial Diffusivity							
	Left Cingulum	-23	-53	11	441	-4.184	-3.154
	Right Dentate Nucleus	5	-59	-18	384	3.272	2.778
Mean Diffusivity							
	Left Cingulum	-23	-53	11	537	-4.162	-3.109
Axial Diffusivity							
	Left Cingulum WM	-18	-54	18	1053	-4.171	-2.928

Regions with significant differences in DTI measures FD, RD, MD, and AD are shown, with their center position, size, peak, and mean z-score. Positive (negative) z-scores correspond to a higher (lower) value of a specific variable in the ADHD group.

### Morphometric data predicting ADHD diagnosis

**Fig 2:**
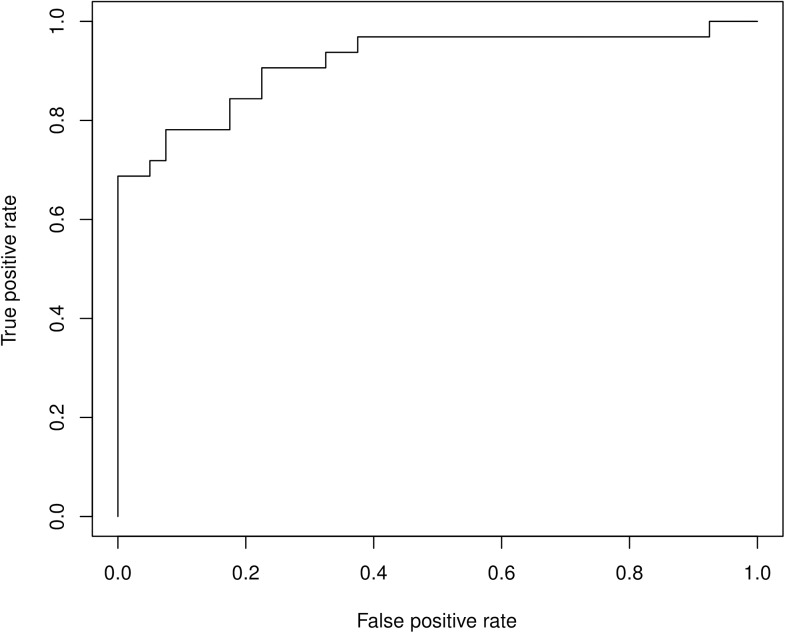
Receiver Operating Characteristics for predicting ADHD based on white matter density. The predictive power for the area under the curve is 0.917.

Overall, a single measure of white matter concentration best predicted ADHD diagnosis (p = 6.18e-06). The receiver operating curve (ROC) analysis revealed that the “area under the curve” (AUC) was found at 0.917, which may be considered as a reasonably high discriminative power of this model. To assess the combined information of six measures (i.e., GM, WM, TBM, FA, RD, MD), PCA was used to reduce the dimensionality to three measures, representing a cumulative variance of 97.5%. Subsequent LDA to develop a simpler classifier correctly predicted an ADHD diagnosis in 83.3% of all cases (see Fig 2). A repeated random sub-sampling cross-validation with a 20% test set yielded a correct prediction rate of 81.4% (95% CI: 0.76–0.86).

## Discussion

An ADHD diagnosis in young adults was associated with decreased grey matter concentration, increased white matter concentration, and decreased shape in widespread areas involving frontal, temporal, striatal, parietal and limbic regions. These widespread macrostructural changes suggest that many of the brain areas associated with an ADHD diagnosis in childhood [16] continue to be affected in young adulthood. More specifically, an ADHD diagnosis in young adults was associated with widespread changes to the maturation of white matter fiber bundles in the brain, such as structural shape changes (incomplete maturation) in the right middle frontal gyrus, right superior temporal gyrus, left middle temporal gyrus, right postcentral gyrus, left posterior insula, and bilateral parahippocampal gyrus. These incomplete maturations in frontal, temporal, parietal, and limbic structures are in agreement with previous findings on cerebral and cerebellar volumetric reductions associated with ADHD in children and adolescents [7, 46].

Our findings revealed reduced gray matter concentrations in the right middle frontal gyrus, right inferior frontal gyrus, right superior temporal gyrus, right middle temporal gyrus, left caudate head, and left parahippocampal gyrus with corresponding white matter increases associated with an ADHD diagnosis in young adults. Thus, frontal, temporal, striatal, parietal, and limbic abnormalities associated with ADHD are not unique to children and adolescents but are also seen in young adults, revealing that the pathophysiology of the disorder seen in childhood carries on into young adulthood. In addition, the reduced grey matter concentrations in the left caudate head in young adults with ADHD suggest that the macrostructural abnormalities in the basal ganglia may normalize at a later stage in adults with ADHD [7, 10, 24].

With regards to microstructural brain abnormalities, our findings corroborate an abnormal cortico-cortical connectivity that develops early and persists into young adulthood [17]. More specifically, increased MD in the left middle temporal gyrus, right internal capsule, and right midbrain suggests reduced white matter density and potential myelin breakdown associated with an adult ADHD diagnosis [4], which is in agreement with in increased MD found in previous studies in children and adolescents with ADHD [47]. Reduced FA and MD were found in the corpus callosum, which corroborates previous findings in children [48, 49] and adults with ADHD [50, 51]. The corpus callosum is central for communicating between different brain areas. Reduced white matter density in this structure may contribute significantly to brain network disturbances associated with ADHD. In addition, decreased FA in the right superior temporal gyrus and both sides of the middle temporal gyrus as well as decreased AD in the occipital lobe and brainstem were associated with an ADHD diagnosis. Low FA and AD values of the white matter may reflect axonal degeneration, and/or less well-organized tracts, and may be induced by a variety of influences [49]. Previous studies in children found increased FA for white matter structures connecting parietal-occipital regions and temporal lobes in children [52, 53]. Our results show that temporal lobes are also affected in size and connectivity in young adults with ADHD.

These DTI findings in young adults with an ADHD diagnosis are in agreement with recent data using whole-brain tractography [28], which revealed widespread disturbances in WM connectivity of children and adolescents involving frontal, striatal, and cerebellar brain regions. Similar to previous research, the dorsal striatum, in particular the putamen, showed regions with reduced FA on the right side and increased RD and MD on both sides including their cortico-striatal connections. The dorsal striatum mediates aspects of decision-making, in particular predicting consequences of goal-directed actions, which are impaired in individuals with ADHD [54]. In addition, increased RD was found in the postcentral gyrus, left middle temporal gyrus, cingulate gyrus, right internal capsule, and right midbrain suggesting decreased myelination or lower fiber density associated with an adult ADHD diagnosis. Decreased connectivity and myelination in these areas may be a stable correlate for ADHD throughout the lifespan [55].

The present findings also corroborated the hypothesis that brain microstructural abnormalities were more associated with child symptoms of ADHD compared to adult symptoms of ADHD. More specifically, dividing into childhood and adult symptoms, the strongest associations between symptoms and morphometric findings were determined by the combination of self-reported childhood symptoms and diffusion-weighted measurements, but less so for childhood symptoms and T1- weighted measures. Although less prominent, a similar pattern was found for the adult symptoms. However, microstructural differences were more strongly associated with childhood than adult symptoms, which may suggest that while microstructural findings are strongly linked to childhood symptoms, such association weakens with adult ADHD symptoms, which may be due to improved structural changes or coping skills.

Intriguingly, childhood symptoms were associated with reduced FA and increased RD in the frontal lobe and putamen/external capsule, which suggests decreased myelination in the frontal–basal ganglia network. Adult symptoms, on the other hand, were associated with reduced FA as well as increased RD and AD in the in the left cingulum and right dentate nucleus, suggesting decreased myelination in these structures in young adults with ADHD. These findings, in part, corroborate previous results of white matter differences associated with symptoms severity [15, 16, 21, 26, 55].

While reduced grey matter in the left caudate was associated with an ADHD diagnosis, reduced FA, as well as increased RD and MD in the left putamen, it was also correlated with child symptoms of ADHD, but not adult symptoms of ADHD. This suggests that disturbances in connections reaching the caudate may be associated with an ADHD diagnosis in young adulthood, whereas reduced FA in the putamen may be an index of symptom severity in childhood.

**Fig 3:**
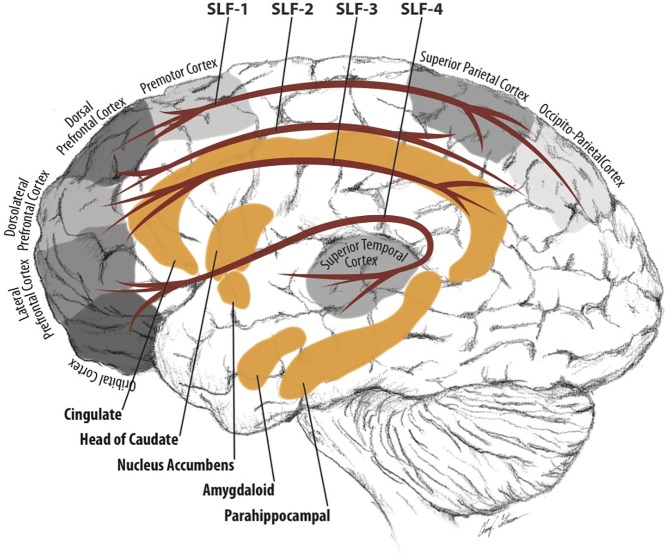
Brain circuitry associated with ADHD.

At the brain circuitry level, the present findings may suggest that the superior longitudinal fasciculus (SLF) and the cortico-limbic associated circuitry are dysfunctional in individuals with ADHD (see Fig 3). The SLF are longitudinal, intra-hemispheric, cortico-cortical bundles of myelinated axons that link the posterior and anterior cortices, including the frontal lobe to the anterior occipital as well as parietal and posterior temporal lobes. The SLF is primarily responsible for the visual and motor coordination, as well as spatial sense, language, and mirror neuron systems. There are several sections of the SLF with different functional contributions to behavior, which associate with the current anatomical findings. More specifically, the dorsal component of the SLF (SLF I) connects the dorsal and medial part of the frontal lobe (i.e., right middle frontal gyrus in Table 2) with the cingulate (Table 3) and cingulum (Table 5) of the superior and medial parietal cortex [56, 57]. As part of the dorsal attention network, the superior parietal cortex has lower connectivity in children with ADHD [58]. However, the SLF I codes primarily for location of body parts in a body-centered coordinate system and regulates higher aspects of motor behavior. Lesions in these regions have been associated with deficits selecting competing motor acts on the basis of appropriate conditional rules [59, 60]. The SLF II connects the middle frontal gyrus (Table 2) with the post central gyrus (Table 4) of the parietal lobe. The SLF II provides information on visual space and visuo-motor function, which may contribute to spatial attention [61–63]. Atypical functional coupling in parts of the SLF II has been associated with ADHD in a functional brain imaging study [64]. Such dysfunctional coupling may result in saccadic abnormalities, which have been found in children and adults with ADHD [65, 66]. The SLF III has been associated with language articulation and connects the rostral part of the inferior parietal lobule with the middle frontal gyrus, the inferior frontal gyrus (Table 2), and the frontal lobe (Table 5) [67]. The arcuate fasciculus and temporo-parietal section of the SLF connect the superior temporal gyrus (Tables 2, 3 and 4) with the prefrontal cortex, which modulates numerous language functions and audio-spatial information [67]. These findings may suggest complex audio-visual dysfunctions associated with ADHD [68–70].

The cortico-limbic associated circuitry connects the amygdala with the thalamus and orbital frontal cortex. It is primarily responsible for emotional learning and behavioral regulation. The present findings involving the caudate head (Table 2), parahippocampal gyrus (Table 2), cingulate gyrus (Table 3), cingulum (Table 5), putamen (Tables 4 and 5), and the external and internal capsule (Tables 2, 3, 4 and 5) suggest a link between cortico-limbic structures and ADHD in young adults. Such link corroborates that dysfunctional motivation, impulsivity, and emotionality may be major contributors to the disruptive behaviors associated with ADHD. A number of studies have shown that ADHD is associated with a motivational deficit due to a dopamine dysfunction in the basal ganglia [71–73]. Such motivational deficit is in agreement with parent reports of children with ADHD who can concentrate during interesting activities but are challenged by mundane and day-to-day tasks [74, 75]. Other studies have documented the comorbidity between ADHD and depression [76, 77]. However, it is yet to be determined if depression is an inherent part of ADHD or perhaps develops secondary in young adults in response to the frustrations associated with not being able to focus as well as risky decision making or lifestyle. In any case, it is unlikely that comorbidities severely influenced the study findings because there were no significant differences in the comorbidities between the ADHD and control groups.

Intriguingly, MRI based data predicted the diagnosis of ADHD correctly in 83% of all the cases. Thus, further research towards defining a MRI-based biomarker is promising, which requires a larger and more diverse patient sample as well as an extended MRI protocol. Future studies could focus on the probability of certain areas and circuitries in predicting an ADHD diagnosis.

### Limitations

The findings have to be interpreted with caution because childhood symptoms were assessed via self-report by young adults, which may be problematic due to memory bias or distortion. In addition, emerging evidence suggests that the ADHD inattentive and ADHD combined subtypes in children may be different disorders based on their unique WM microstructure [78], which was not taken into consideration in this study. Larger studies are needed to develop a better understanding of the anatomical differences between ADHD subtypes. Furthermore, while previous morphometric and DTI studies on white/gray matter integrity in adults with ADHD revealed deficits in structural connectivity, they may be not unique to ADHD [79]. Thus, more research is necessary to determine whether the morphometric findings presented here are specific to ADHD.

## Conclusion

An adult ADHD diagnosis and in particular child symptoms were associated with widespread micro- and macrostructural changes in the frontal, basal ganglia, anterior cingulate, temporal, and occipito-parietal regions in young adults with ADHD. The associations between brain structure and ADHD symptoms in childhood were more widespread and prominent (i.e., number, size, and z-scores of significant brain regions) than ADHD symptoms in adulthood. In addition, the analysis of the diffusion-weighted measures yielded stronger results (in terms of number, size, and z-scores of significant regions) than the measures derived from T1-weighted data. Thus, in relation to the diagnosis and symptom scores, changes in the microstructural properties of white matter fiber tracts appear to be stronger and more extensive than macrostructural differences. This highlights the importance of the microstructural architecture in the pathophysiology of ADHD.

The overall morphometric findings predict the ADHD diagnosis in 83% of cases, which demonstrates sufficient sensitivity to aid in the clinical assessment and potential as a biomarker for ADHD in young adults. At the brain circuitry level, the involvement of the SLF and cortico-limbic areas may suggest complex audio-visual, motivational, and emotional dysfunctions associated with ADHD in young adults, which could be investigated in future studies. Investigating the SLF and cortico-limbic circuitries may be an interesting and novel research direction, which may lead to a better understanding of the underlying mechanisms of ADHD and the development of novel treatments that target audio-visual, motivational, and emotional dysfunctions.

## Supporting information

S1 Template Study Specific Anatomical Template. (GZ)

S1 Map Grey Matter Map-Diagnosis. (GZ)

S2 Map White Matter-Diagnosis. (GZ)

S3 Map JAC Map-Diagnosis. (GZ)

S4 Map Fractional Anisotropy Map-Diagnosis. (GZ)

S5 Map Radial Diffusivity Map-Diagnosis. (GZ)

S6 Map Mean Diffusivity Map-Diagnosis. (GZ)

S7 Map Axial Diffusivity Map-Diagnosis. (GZ)

S8 Map Fractional Anisotropy Map-Childhood Symptoms. (GZ)

S9 Map Radial Diffusivity Map-Childhood Symptoms. (GZ)

S10 Map Mean Diffusivity Map-Childhood Symptoms. (GZ)

S11 Map Axial Diffusivity Map-Childhood Symptoms. (GZ)

S12 Map Fractional Anisotropy Map-Adult Symptoms. (GZ)

S13 Map Radial Diffusivity Map-Adult Symptoms. (GZ)

S14 Map Mean Diffusivity Map-Adult Symptoms. (GZ)

S15 Map Axial Diffusivity Map-Adult Symptoms. (GZ)
